# Editorial: Women in pharmacoepidemiology: 2021

**DOI:** 10.3389/fphar.2023.1278768

**Published:** 2023-09-12

**Authors:** Luciane Cruz Lopes, Daniela C. Moga, Tatiane Da Silva Dal Pizzol, Natasa Gisev, Bita Mesgarpour, Anick Bérard

**Affiliations:** ^1^ Pharmaceutical Science Graduate Course, University of Sorocaba, Sorocaba, Brazil; ^2^ Department of Pharmacy Practice and Science, College of Pharmacy, University of Kentucky, Lexington, KY, United States; ^3^ Graduate Program of Epidemiology, School of Medicine, Federal University of Rio Grande do Sul (UFRGS), Porto Alegre, Brazil; ^4^ Department of Production and Control of Medicines, School of Pharmacy, UFRGS, Porto Alegre, Brazil; ^5^ National Drug and Alcohol Research Centre, UNSW Sydney, Sydney, Australia; ^6^ National Institute for Medical Research and Development, Tehran, Iran; ^7^ CHU Sainte Justine Research Center, Faculty of Pharmacy, University of Montreal, Montréal, QC, Canada

**Keywords:** women, pharmacoepidemiolgy, public healh, real world data (RWD), medicine

This is the first Research Topic offering the opportunity to promote the work of women scientists at different stages of their careers, worldwide, and in all areas of pharmacoepidemiology. This Research Topic contains 11 studies led by women from different parts of the world, including five studies from Brazil, three from Canada, one from the United States, one from Switzerland and one from Romania. The work presented here highlights the diversity of research carried out across the breadth of pharmacoepidemiology research and presents advances in theory and methodology with applications to compelling problems. This current Research Topic includes studies related to chronic health conditions, including cardiovascular, renal, respiratory, and cancer diseases.

An important theme present in this Research Topic is the use of real world data (RWD) to generate evidence for decision-making. RWD has gained significant attention in the field of research as it provides a valuable data source beyond traditional clinical trials and lab-based experiments. RWD can come from various sources, including but not limited to healthcare administrative data, hospitalization databases, electronic health databases, surveys, data from government agencies and others. Healthcare administrative data refers to the information collected and maintained by healthcare organizations, insurers, and government agencies for the purpose of managing, tracking, and reimbursing various aspects of healthcare services. An interesting scoping review (Bukhtiyarova et al.) was conducted to explore the current state of existing research according to the application of Artificial intelligence (AI) to healthcare administrative data, including those involving medications. The application of AI to healthcare administrative data is heterogeneous in terms of areas of interest and methods. One of the points highlighted by the authors is that AI can significantly improve research on the utilization of healthcare administrative data. This phenomenon can be explained by the accumulation of large volumes of this type of data, improved access to such databases for AI researchers, further development of AI methods, improved computational capacities, and increased funding of interdisciplinary projects. The authors found that many studies were focused on data from hospitals and emergency departments which can be explained by better accumulation of data by large hospitals that are often affiliated to universities, and their better accessibility for AI research. The most popular health areas for the application of AI include the prediction of health outcomes and the handling of large health datasets. These findings point to the need to improve data collection and accessibility for scientific research of outpatient data. Three studies (Barbosa et al., Frent et al., Okuyama et al.) used data from pharmacovigilance databases to generate safety data. Okuyama et al. analyzed cross-referenced information from three different healthcare administrative databases: official death records between 1996 and 2019 from the Mortality Information System in Brazil, hospitalizations between 2008 and 2020 from the Hospital Information System, and cases of poisonings between 2017 and 2020 in health services that have been notified to the National System for Vigilance of Notifiable Diseases. The analysis shows that the reports were more frequent among adults, particularly women, and due to accidents, with exposure to paracetamol identified as a concern for preventable intoxications, hospitalizations and deaths. Barbosa et al. identified early safety signals of antibacterial agents to support pharmacovigilance systems using data from a Brazilian database (Vigimed/VigiFlow) from December 2018 to December 2021. Vancomycin was the most reported antibiotic, followed by ceftriaxone and piperacillin and tazobactam. Three serious events were associated with ceftazidime and avibactam, a new drug in the Brazilian market. Frent et al. performed a descriptive analysis of cases of acute renal failure and nephrolithiasis reported from Sodium–Glucose Co-Transporter 2 (SGLT2) inhibitors in VigiBase to September 2021. The vast majority of acute renal failure and nephrolithiasis reports were considered serious. Canagliflozin was the gliflozin most involved in cases of acute renal failure and nephrolithiasis.

Healthcare records are another important source of real world data. Healthcare records play a crucial role in generating RWD in the field of healthcare and medical research. One way that healthcare records contribute to generating real-world evidence is through observational studies using de-identified patient data from healthcare records. A cross-sectional database study in Switzerland (Rachamin et al.) used medical records to determine patient age- and sex-specific prevalence rates of polypharmacy, and rates of prescribing of the most frequently used medication classes; they also explored practitioner variability in prescribing practices in Swiss general practitioners. The prevalence of polypharmacy in the adult Swiss general practice population was determined to be 24%, increasing with age: from 6% in patients aged 18–40 years to 65% in patients aged 81–92 years. Women had higher rates of polypharmacy than men. The difference was more pronounced at younger ages, and interestingly, hormonal contraceptives did not relevantly contribute to the difference. The most clear drivers of the differing rates of polypharmacy in the younger population were higher prescription rates of vitamins, mineral supplements, and antianemic preparations in female patients. Men were more often prescribed agents targeting the cardiovascular system (such as antihypertensive agents, antithrombotic agents, and lipid modifying agents), whereas most other medications were more often prescribed to women. A cohort study (Gorgui et al.) undertaken in Canada involving the linkage of three Quebec databases was undertaken to quantify the risk of babies being born small for gestational age (SGA) and very small for gestational age (VSGA) with the use of medically assisted reproduction. While no association was observed between medically assisted reproduction and SGA or VSGA in the study population; medically assisted reproduction was associated with an increased risk for SGA among preterm pregnancies; no increased risk of SGA was observed in term pregnancies. Another cohort study (Oh et al.) examined the association between gabapentin use and neurocognitive changes in older adults using the National Alzheimer’s Coordinating Center Uniform Data Set (NACC UDS). The NACC UDS has rich data and is an example of how building a database with real world data focused on answering research questions can contribute to decision making in health. This data set provided a greater sample size compared with previous studies in this population and was able to measure the association between gabapentin initiation and neurocognition with various clinically relevant outcomes. The results provided evidence that gabapentin was associated with increased odds of global cognitive decline, functional status decline, and motor function change in the 2 years following gabapentin initiation. Considering that gabapentin may block calcium channels in the brain, it was hypothesized that it would have a neuroprotective effect, but these findings did not support this assumption. The authors concluded that further experimental studies are needed to examine the mediators of gabapentin use and neurocognitive changes.

On the other hand, systematic reviews with meta-analyses (SR-MA) also play a crucial role in informing healthcare decisions, policy-making, and advancing scientific knowledge. Two systematic reviews and meta-analyses published in this Research Topic were designed to complement the knowledge obtained from randomized control trials and observational studies of primary data to evaluate the real-world effectiveness of medications. These reviews (Castro et al., Queiroz et al.) summarized the evidence produced from observational studies involving administrative databases of different drug classes of disease-modifying drugs (DMARDs) in patients with rheumatoid arthritis (RA). The meta-analysis by Queiroz et al. did not suggest there is an increased risk of adverse events associated with the use of biologics for the treatment of RA, indicating a lower risk of cardiovascular events with abatacept than tumor necrosis factor inhibitors (TNFi) (low to very low certainty of the evidence). The meta-analysis by Castro et al. synthesized data on 182,098 RA patients across 21 studies included. They concluded that biologics effectively treat patients with RA, with higher effectiveness for non-TNFi and Janus kinase inhibitors (JAKi) than with TNFi. A policy brief is another type of evidence synthesis combining research evidence specific to stakeholders’ contextual knowledge. The policy brief from Fulone et al. identified evidence-based strategies to improve adherence to preventive measures against COVID-19 at the community level. Three evidence-based strategies were identified: i) Risk communication; ii) Health education to the general public, and iii. Financial support and access to essential supplies and services. The evidence showed that an increase in knowledge, transparent communication, and public awareness about the risks of COVID-19 and the benefits of adopting preventive measures result in changes in people’s attitudes and behavior, which can increase adherence. These strategies can guide future actions and the formulation of public policies to improve adherence to preventive measures in the community in future epidemics. Finally, this Research Topic also includes a protocol on a scoping review (Leal et al.) describing the methodology for assessing the available literature on emulation target trials to study outcomes in women exposed to medications in the preconception, perinatal, or postpartum periods.

To conclude, this first Research Topic on Women in Pharmacoepidemiology highlights the range of robust research that is being performed by women in science, and pharmacoepidemiology in particular, across the globe today. Research is varied, and complementary, and is being undertaken across the life course, from conception to adulthood. However, the lack of true global representation of women in science is a significant issue that has persisted for many years and requires a multi-faceted approach to address the issue. Efforts to increase the representation of women in science should be a priority for governments, educational institutions, scientific organizations, and society as a whole. By breaking down barriers and creating more inclusive environments, we can unlock the full potential of women in advancing scientific discovery and innovation. We need to do better in the future to promote the work of women scientists all over the world. It is with great pleasure that we are presenting *Women in pharmacoepidemiology: 2021*.

